# Higher Potassium Intake and Lower Sodium Intake May Help in Reducing CVD Risk by Lowering Salt Sensitivity of Blood Pressure in the Han Chinese Population

**DOI:** 10.3390/nu14204436

**Published:** 2022-10-21

**Authors:** Yunyi Xie, Han Qi, Wenjuan Peng, Bingxiao Li, Fuyuan Wen, Fengxu Zhang, Ling Zhang

**Affiliations:** 1 Department of Epidemiology and Health Statistics, School of Public Health, Capital Medical University, Beijing 100069, China; 2 Beijing Municipal Key Laboratory of Clinical Epidemiology, Capital Medical University, Beijing 100069, China

**Keywords:** cardiovascular disease risk, salt sensitivity, twenty-four-hour urinary sodium:potassium ratio, mediation

## Abstract

Sodium (Na) reduction with a parallel supplemental potassium (K) intake can prevent cardiovascular diseases (CVDs). The relationship of the urinary Na/K ratio and salt sensitivity of blood pressure (SSBP) with CVDs is not clearly explained. We assumed that the SSBP mediates the relationship between the Na/K ratio and CVDs. In total, 2055 subjects who had 24 h urine collected and SSBP determined were included in this study. CVD risk was estimated using the China-PAR equation. MediationMultivariate logistic regression was used to explore the associations between the Na/K ratio or SSBP with CVD risk. Mediation analysis using a logistic regression model was performed. Both the urinary Na/K ratio and SSBP were related to the estimated CVD risk (*p* < 0.05). The mediation analysis found that SSBP mediated approximately 12% of the association between Na/K ratio and CVD risk. Our findings indicate that higher K intake and lower Na intake may help in preventing CVD risk by reducing SSBP risk in individuals with normotension or stage-one hypertension.

## 1. Introduction

Cardiovascular diseases (CVDs) are the number one determinant of death globally, and caused approximately 17.9 million human deaths in 2016, or about 31% of deaths worldwide [1]. Studies have found that high-salt diets can lead to vascular remodeling, increase the burden on the heart, and increase the risk of stroke [2], and sodium intake is one of the main dietary risk factors of CVDs [3]. Population-wide sodium reduction can control blood pressure (BP) and thus mitigates the burden of CVDs [4].

Urinary sodium (Na) and potassium (K) excretion are related to BP and CVDs [5,6]. Recent studies have shown that CVDs are associated with Na and K balance and a high dietary Na/K ratio can raise the risk of CVDs [7]. Studies have reported that more urinary sodium excretion was individually associated with a higher risk of CVDs among the Chinese population [8,9]. However, few studies have reported an association between CVD risk and either urinary potassium excretion or the Na:K ratio in China. Moreover, individuals display discrepancy in their BP in response to salt intake. Salt sensitivity of blood pressure (SSBP) is viewed as a quantitative trait in which the BP of some individuals of the population exhibit changes in relation to changes in salt ingestion [1]. Individuals can also be divided into salt-sensitive (SS) or salt-resistant (SR) according to the salt-induced change in BP [1]. The well-known GenSalt research shows that about 32% Chinese adults are SS [10]. A 7.3-year prospective cohort study with a relatively small hypertensive population found that the incidence of all kinds of cardiovascular events in the SS group was significantly higher than that of the SR group [11]. More knowledge about the role of SSBP as a risk factor for CVDs will help to reduce their burden, and further research on it is needed. Furthermore, Morris Jr. suggested that SS occurs when dietary potassium is lightly deficient in most normotensive black men and increasing dietary potassium can reduce the frequency of salt sensitivity [12]. Some animal studies showed that the dietary Na/K ratio can be a determinant of salt sensitivity [13,14]. The Na/K ratio could be a pivotal contributor to shared pathological pathways linking SSBP and CVD risk. However, the association of the SSBP with the Na/K ratio and CVD risks has not been well-clarified.

Accordingly, the present study is based on previous studies’ results and explores the relationship between the Na/K ratio and CVDs while elucidating the possible contribution of SSBP to the Chinese population. The China-PAR equation is a sex-specific algorithm used to assess the 10-year risk for atherosclerotic cardiovascular disease (ASCVD) [15]. Present recommendations for the prevention of CVD risk factors in clinical practice primarily concentrate on the evaluation of the patient’s overall disease risk rather than any specific risk factor [16]. Therefore, the China-PAR equation, which represents the risk for all kinds of CVDs, can assess the total risk of CVDs. Our first hypothesis was that both SSBP and the Na/K ratio would be related to the 10-year risk for CVDs. Considering that the Na/K ratio may be related to SSBP, we further investigated whether the association between the Na/K ratio and CVDs could be explained by SSBP. Our second hypothesis was that associations between the Na/K ratio and the estimated CVD risk would be partially explained by SSBP. Finally, we used a mediation model to explore whether SSBP statistically mediates the association between the Na/K ratio and CVD risk.

## 2. Materials and Methods

### 2.1. Study Design and Sample Collection

A total of 2163 unrelated subjects were included in the Systems Epidemiology Study on Salt Sensitivity (EpiSS). This program was implemented between July 2014 and July 2016 with the aim of the identification of environmental and genetic risk factors for SSBP in a Northern Chinese population. The protocol for this program has been illuminated in detail previously [17]. Briefly, subjects were included in this study if they met the following criteria: aged 35–70 years old; unrelated Chinese Han people; long-term residents for more than 5 years; (3) normal blood pressure or patients with stage-one hypertension (SBP to 140 to <160 mmHg and (or) DBP to 90 to <100 mmHg, based on the defined standard of the 2010 Chinese hypertension guidelines [18]. Participants were excluded if they were pregnant women, or had severe CVDs, kidney disease, liver disease, or cancerous tumors. SSBP was determined using the modified Sullivan’s acute oral saline load and diuresis shrinkage test (MSAOSL-DST). Demographic characteristics, lifestyle risk factors, medical case history, medical family history, smoking and drinking habits, and body measurement information were collected. The fasting blood glucose (FBG) was tested using the hexokinase/glucose-6-phosphate dehydrogenase approach. Lipid profile, including total cholesterol (TC), triglyceride (TG), low-density lipoprotein cholesterol (LDL-C), and high-density lipoprotein cholesterol (HDL-C), was measured using an enzymatic approach. The body mass index (BMI) was defined as weight in kilograms divided by the square of height in meters. Waist circumference (WC) was recorded at one centimeter above the navel at minimal respiration. The study was approved by the Capital Medical University ethics committee (no. 2013SY22) and was registered in the WHO International Clinical Trials Registry Platform (no: ChiCTR-EOC-16009980). All subjects signed their informed consent before participating in this research.

### 2.2. Assessment of SSBP

The evaluation of SSBP was conducted using MSAOSL-DST, the details of which have been previously published [17,19]. All the participants were asked to stop taking antihypertensive drugs for at least twenty-four hours before the test. Each subject took an oral administration of 1000 mL of 0.9% saline solution within 30 min and 40 mg furosemide after two hours of the saline loading. After a five-minute break in the sitting position, automatic sphygmomanometers (Omron HEM-7118, Kyoto, Japan) were used to measure BP two times at min intervals. The mean value represented the final BP value. The BP was tested at the following three time points: before saline loading, two hours after the saline loading, and two hours after the oral furosemide intake. Systolic BP (SBP) and diastolic BP (DBP) were measured. We used the following formula for the mean arterial pressure (MAP): MAP = (SBP + 2 × DBP)/3 [20]. MAP change 1 was defined as MAP after acute salt loading for two hours minus MAP before acute salt loading, and MAP change 2 was defined as MAP after diuresis shrinkage for two hours minus MAP before diuresis shrinkage. MAP change 1 and MAP change 2 were used to represent SSBP in this study.

### 2.3. Measurements for Sodium and Potassium Excretion

All subjects were requested to throw out the first-morning urine sample and then collect total urine samples from that moment on for 24 h [21]. Each subject was provided a 5 L plastic bucket with a lid to gather 24 h urine samples. The 24 h urine sample was collected 7 days after the SSBP assessment with a normal diet. A urine volume of below 500 mL was deemed as an invalid sample [22]. 24 h urine Na (24hUNa) and K (24hUK) concentrations were measured using ion-selective electrode techniques and analyzed with a Cobas C8000 (Roche, Basel, Switzerland). The 24 h urinary Na/K ratio (24hUNa/K) was determined via dividing the moles of Na by the moles of K.

### 2.4. Measurements for 10-Year CVD Risk

The China-PAR equation is a sex-specific algorithm that serves as an assessment tool for the 10-year risk for atherosclerotic cardiovascular disease (ASCVD) [15]. The China-PAR equation is computed based on age, sex, smoking, SBP, TC, HDL-C, hypertensive treatment status, diabetes, WC, geographic region, urbanization (only for men), and family history of ASCVD (only for men). Individuals were divided into two subgroups according to their 10-year CVD risk as estimated using the China-PAR equation: <10% (low-moderate CVD risk group) and ≥10% (high CVD risk group) [23]. The 10-year CVD risk was used as the binary outcome variable (low-moderate CVD risk group versus the high CVD risk group) in this study.

### 2.5. Statistic Methods

The general characteristics data are displayed as numbers with percentages for categorical variables and as means with standard deviations (SDs) or medians with an interquartile range (IQR) for continuous variables. Student’s *t*-test was used to check the differences in the continuous variable with normal distribution between the two groups. The Wilcoxon rank-sum nonparametric test was conducted to examine continuous variables with non-normal distribution or rank variables. Categorical data between the two groups were analyzed utilizing the chi-squared test. Log transformations were used to transform skewed sodium and potassium data. To analyze the associations between SSBP or Na/K ratio with CVD risk, multivariate linear regression and multivariate logistic regression were performed. For ease of explanation, NA, K, and Na/K ratios were all categorized into quartiles.

**Figure 1:**
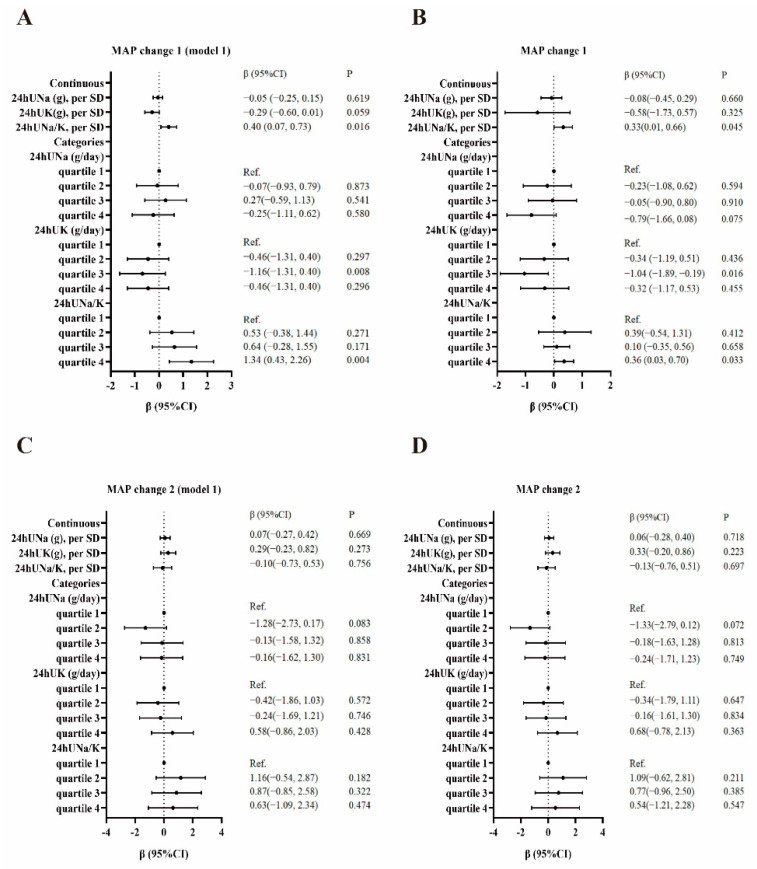
Association between sodium, potassium, sodium: potassium ratio, and SSBP. MAP change 1 as the outcome (**A**). MAP change 1 as the outcome and adjusted for age, gender, BMI, FBG, and baseline MAP (**B**). MAP change 2 as the outcome (**C**). MAP change 2 as the outcome and adjusted for age, gender, BMI, FBG, and baseline MAP (**D**).

To examine whether the SSBP as a mediator was involved in the association between the Na/K ratio and CVD risk, we utilized the common protocol (Figure 1A) [24]. Considering the relationship between SSBP or the Na/K ratio and CVD risk has been examined, we analyzed whether SSBP was a possible mediator of this path. Continuous SSBP, the Na/K ratio, and CVD risk were utilized for the mediation model adjusted for baseline MAP. The PROCESS macro v2.16.3 for SPSS was utilized to test the mediation path. Bootstrapping was used to test mediation signification [25]. Model 4 (used for simple mediation models) from the PROCESS macro was utilized with 5000 bootstraps to compute the direct and indirect effect between the Na/K ratio, SSBP, and CVD risk. *p* ≤ 0.05 was considered significant. The statistical test was operated in IBM SPSS Statistics 24.0 software (SPSS, Chicago, IL, USA).

## 3. Results

### 3.1. Study Population and Baseline Characteristics

The baseline characteristics of all participants are summarized in Table 1. A total of 2055 participants (27% male, medium age of 59 years), including 659 (32.1%) low-moderate CVD risk subjects and 1396 (67.9%) high CVD risk subjects, were recruited for the study. The medium 24hUNa was 3.15 g, 24hUK was 1.67 g, and 24hUNa/K was 3.17. The high CVD risk group was demonstrated to have higher 24hUNa levels (*p* < 0.05).

### 3.2. Association of SSBP with the 10-Year CVD Risk

We conducted logistic regression analyses to evaluate the associations of SSBP (MAP change 1 and MAP change 2) with the 10-year CVD risk (Table 2). The MAP change 1 as a continuous variable was positively associated with the 10-year CVD risk (multivariate *p* < 0.001), while the MAP change 1 showed no significant association (multivariate *p* = 0.596) after being adjusted for 24hUNa/K. Compared with cases in the lowest quartile of MAP change 1, those in the highest quartile were associated with a higher 10-year CVD risk (OR = 1.70, 95% CI: 1.23, 2.36, *p* = 0.001). After adjustment for 24hUNa/K, compared with cases in the lowest quartile of MAP change 1, those in the highest quartile were associated with a higher 10-year CVD risk (OR = 1.69, 95% CI: 1.22, 2.33, *p* = 0.002). Compared with cases in the lowest quartile of MAP change 2, those in the second quartile were associated with a lower 10-year CVD risk (OR = 0.71, 95% CI: 0.53, 0.96, *p* = 0.025). After adjustment for 24hUNa/K, compared with cases in the lowest quartile of MAP change 2, those in the second quartile were associated with a lower 10-year CVD risk (OR = 0.72, 95% CI: 0.54, 0.96, *p* = 0.027). In total, these results suggest that SSBP is a risk factor for CVDs. The results of the association between SSBP (MAP change 1 and MAP change 2) with the 10-year CVD risk obtained from linear regression analysis are similar to the above results (Supplementary Table S1).

### 3.3. Association of Sodium, Potassium, and Sodium:Potassium Ratio with the 10-Year CVD Risk

As shown in Table 3, a multivariate logistic regression analysis was conducted to measure the associations of 24hUNa, 24hUK, and the 24hUNa/K with the 10-year CVD risk. After adjusting for the MAP change 1, the 24hUNa and 24hUNa/K as continuous variables were positively associated with the 10-year CVD risk (multivariate *p* < 0.001 and multivariate *p* = 0.025, respectively). No significant association was observed between 24hUK and the 10-year CVD risk (*p* = 0.076).

We found that the odds of having a high CVD risk were increased for those in the second, third, and fourth quartile of the 24hUNa (OR_2nd_ = 1.62, 95% CI: 1.25, 2.09; OR_3rd_ = 1.67, 95% CI: 1.29, 2.16; OR_4th_ = 1.98, 95% CI: 1.52, 2.58; *p* for trend <0.001), compared with cases in the lowest quartile after being adjusted for the MAP change 1. The odds of having a high CVD risk were increased for those in the third and fourth quartile of the 24hUK (OR_3rd_ = 1.43, 95% CI: 1.08, 1.89; OR_4th_ = 1.46, 95% CI: 1.10, 1.94; *p* for trend = 0.004), compared with cases in the lowest quartile after being adjusted for the MAP change 1. The odds of having a high CVD risk were increased for those in the third and fourth quartile of the 24hUNa/K (OR_2rd_ = 1.45, 95% CI: 1.09, 1.92; OR3rd = 1.48, 95% CI: 1.11, 1.96; *p* for trend = 0.19), compared with cases in the lowest quartile after being adjusted for the MAP change 1. The results of the association between 24hUNa, 24hUK, and the 24hUNa/K with the 10-year CVD risk obtained by linear regression analysis are similar to the above results (Supplementary Table S2).

### 3.4. Association of Sodium, Potassium, and Sodium:Potassium Ratio with the SSBP

We conducted univariate and multivariate linear regression analyses to evaluate the associations of 24hUNa, 24hUK, and 24hUNa/K with the SSBP (MAP change 1 or MAP change 2). The results are shown in Figure 1. The 24hUNa/K as a continuous variable was positively associated with MAP change 1 before (β = 0.40, 95% CI: 0.07, 0.73) and after (β = 0.33, 95% CI: 0.01, 0.66) being adjusted for age, gender, BMI, FBG, and baseline MAP. No significant associations were observed between 24hUNa and 24hUK as continuous variables with MAP change 2 (*p* > 0.05). In the univariate linear regression model, compared with cases in the lowest quartile of 24hUK, those in the third quartile were associated with a lower MAP change 1 (β = −1.16, 95% CI: −1.31, 0.40). Compared with cases in the lowest quartile of 24hUNa/K, those in the fourth quartile were associated with a higher MAP change 1 (β = 1.34, 95% CI: 0.43, 2.26). After adjustment for age, gender, BMI, FBG, and baseline MAP, compared with cases in the lowest quartile of 24hUK, those in the third quartile were associated with a lower MAP change 1 (β = −1.04, 95% CI: −1.89, −0.19). Compared with cases in the lowest quartile of 24hUNa/K, those in the fourth quartile were associated with a higher MAP change 1 (β = 0.36, 95% CI: 0.03, 0.70).

Further stratified analysis was performed to test the association between the 24hUNa, 24hUK, and 24hUNa/K and SSBP in individuals with or without hypertension. As shown in Supplementary Table S3, for normotension subjects, the 24hUNa/K as a continuous variable was significantly associated with MAP change 1 after adjusting for age, gender, BMI, FBG, and baseline MAP (β = 0.58, 95% CI: 0.010, 1.15). Compared with cases in the lowest quartile of 24hUNa/K, those in the fourth quartile were associated with a higher MAP change 1 (β = 0.52, 95% CI: 0.11, 0.94). Compared with cases in the lowest quartile of 24hUK, those in the third quartile were associated with a lower MAP change 1 (β = −0.65, 95% CI: −1.17, −0.12). For hypertension subjects, compared with cases in the lowest quartile of 24hUNa/K, those in the second quartile were associated with a higher MAP change 2 (β = 1.85, 95% CI: 0.24, 3.45).

### 3.5. Mediation Effect of SSBP between Urinary Sodium:Potassium Ratio and the 10-Year CVD Risk

**Figure 2:**
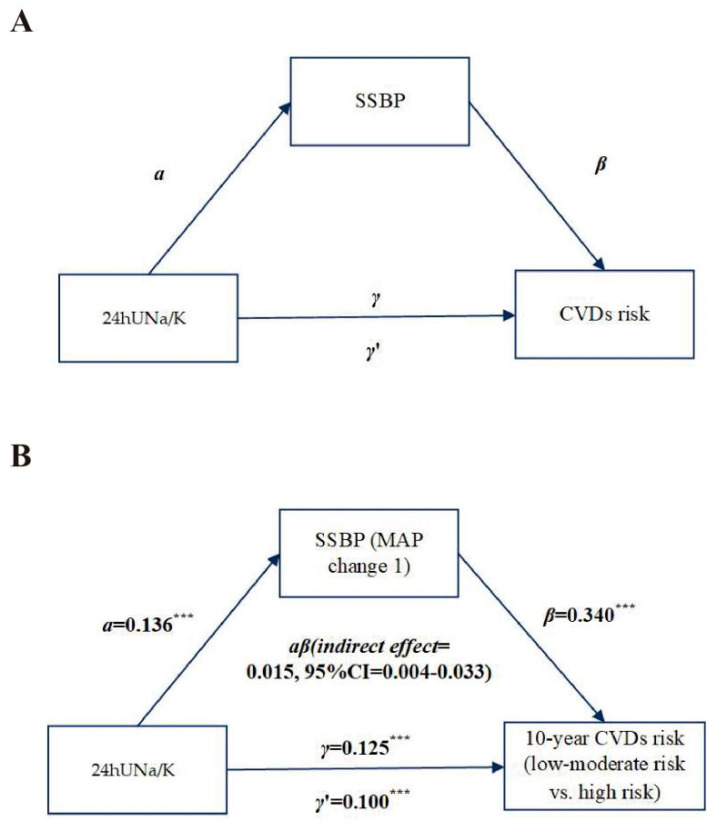
Mediation effect of SSBP between Na/K ratio and 10-year CVD risk. Illustration diagram of regression analysis steps necessary to conduct a simple mediation model (**A**). Mediation effect of SSBP between 24hUNa/K and the 10-year CVD risk (**B**). Footnote: unstandardized regression coefficients (*τ*, *τ*′, α, and β) were used in the model. The regression coefficient between 24hUNa/K and the 10-year CVD risk, controlling for SSBP, is indicated as *τ*’ (direct effect) while the regression coefficient between 24hUNa/K and the 10-year CVD risk only (total effect) is indicated as *τ*. ***: *p* < 0.001.

Based on the above results, we analyzed whether SSBP is a mediator of the relationship between the 24hUNa/K and the 10-year CVD risk adjusted for baseline MAP as a covariate (Figure 2). A significant total effect of the 24hUNa/K on the 10-year CVD risk was found (path γ = 0.13, *p* < 0.001). MAP change 1 was also significantly associated with the 10-year CVD risk (path β = 0.340, *p* < 0.001). The 24hUNa/K was a significant predictor of SSBP (path α = 0.14, *p* < 0.001). Furthermore, mediation analysis showed a significant indirect effect of MAP change 1 on the 10-year CVD risk (indirect effect: αβ = 0.02, 95% CI: 0.000–0.03). The direct effect of the 24hUNa/K on the 10-year CVD risk was also significant, indicating independence from SSBP (path γ’ = 0.10, *p* < 0.001). These findings suggest that the SSBP partly mediated the relationship between the 24hUNa/K and the 10-year CVD risk; the mediating proportions were 12%. However, we found no significant mediating effects of SSBP, from the 24hUNa or 24hUK, on the 10-year CVD risk (*p* _indirect effect_ > 0.05).

## 4. Discussion

The results of our analyses confirm our two hypotheses. People with elevated SSBP or 24hUNa/K were more likely to have a high CVD risk in normotension or stage-one hypertensive individuals. Additionally, by connecting SSBP and 24hUNa/K in the same model, our results suggest new evidence that SSBP partly mediates the relationship between 24hUNa/K and CVD risk.

The results from a smaller, dietary-salt-loading SSBP study have indicated similar outcomes for SSBP and CVDs. Jing Chen suggested that SSBP had a positive association with metabolic syndrome, which is associated with an increased risk of CVDs [26]. Weinberger carried out a 27-year follow-up study that indicated that SSBP was associated with increased mortality independent of BP [27]. Moreover, SSBP is associated with many CVD risk factors. The association between low birth weight, which can predict CVDs in adult life and SSBP, was significant, and the relationship was independent of creatinine clearance [28]. Furthermore, SS individuals are more insulin-resistant than SR individuals [29,30], and they display lower nocturnal BP dipping, which is associated with poorer CVD prognosis [31]. However, few studies examine the relationship between SSBP and CVDs in Asia, particularly in China. In our study, we provide novel evidence that SSBP is associated with the 10-year CVD risk.

Our results of a positive relationship between the 24hUNa/K and CVD risk have been suggested in other studies [32,33,34]. Nonetheless, dose–response relationships between Na and K intake and the Na/K with CVDs have not been fully explored [35] (Hooper LBartlett 2002). The interaction of Na and K may have an important role in the development of hypertension and the progress of CVDs [36] (Nancy R. Cook, 2009). High Na intake may lead to an increased CVD risk through the declining activity of the renin–angiotensin–aldosterone system (RAAS) and rising cardiac output [37]. The RAAS is an important regulator of blood volume and systemic vascular resistance, which is related to the pathogenesis of hypertension [38] (Roberto Ferrari, 2013). Higher cardiac output can lead to hypertension [39]. The effect of high Na on BP is affected by diet composition, particularly the K content. High dietary K is related to the decline in BP in the presence of a high K diet [40]. One study conducted on Iranian participants suggested no relationship between dietary intakes of sodium and potassium and CVDs; however, participants with the highest compared to lowest tertile of Na/K ratio had an enhanced risk of CVDs [33]. Other studies showed that the dietary Na/K ratio was a significant risk factor for CVDs and all-cause mortality [32] and associated with several CVD risk factors among Japanese people [34]. Several studies indicated that a high dietary 24hUNa/K was a risk factor for diabetes, and associated with high insulin resistance [39,41]. Shufa Du suggested that the Na/K ratio and incidence of hypertension have a positive dose–response relationship in Chinese adults [42]. However, few studies have researched the relationship between the Na/K ratio and CVD risk among the Chinese population. Our study showed that 24hUNa/K was significantly associated with CVD risk among the Chinese population.

Consistent with our second hypothesis, our results indicate that SSBP (MAP change 1) partly mediates the relationship between the Na/K ratio and CVD risk. The antagonistic effect of sodium and potassium is mainly reflected in renal excretion. The K may be involved in the antihypertensive effect through the direct natriuretic effect, which converts SS hypertension to SR hypertension [43]. Sodium load will cause increased urinary potassium excretion, resulting in conditional potassium deficiency. Increasing potassium intake can prevent the salt-induced increase in BP by promoting sodium excretion and curbing volume expansion [44]. Plus, Na excess and K deficit can increase sympathetic stimulation [45], and a lightly insufficient dietary potassium intake can increase vasopressor responsiveness to sympathetic nervous stimulation caused either by internal factors or environmental factors [12]. In certain rodent models, sympathetic overactivity may induce SS [46,47]. The above evidence supports our finding that SSBP was a significant mediator of the relationship between the 24hUNa/K and CVD risk. The mediation effect of SSBP (MAP change 2) between the Na/K ratio and CVD risk was not overserved, and no significant association was found between MAP change 2 and the Na/K ratio. This may be due to the different mechanisms of the human physiological response to salt loading and salt depletion. Sodium loading restraints and sodium depletion arouse the RAAS system stimulating changes in BP during changes in sodium intake [37]. Our previous study showed that genes involved in the process of rising vascular reactivity were associated with MAP change 1 while genes associated with MAP change 2 were related to decreasing vascular reactivity or renal sodium transport function [48].

We identify several limitations. First, differing approaches to distinguishing SSBP have been established. Nevertheless, there is no universal approach. In this study, SSBP was determined using MSAOSL-DST. This method is a modification of Sullivan’s method, which has been utilized in several types of research among the Chinese population [19]. Second, while representative of the Chinese population, our study had a comparatively small sample size. However, the assessment of SSBP is challenging to carry out, and it is not easy to measure SSBP in community-based populations. Third, although a causal relationship was suggested by the mediation analysis, considering that the data in this study come from a cross-sectional study, only an association can be concluded. Additional study is necessary to examine whether this is replicable and whether the experimental study can offer stronger evidence of causality. Fourth, a single 24 h urine collection may not show an individual’s long-term daily Na intake, although 24 h Na excretion is the recommended approach for assessing population mean Na intake [21,49]. Important strengths of our study include our participants being selected from the community, which can minimize selection bias, and the relatively large sample size for the SSBP study. We believe this study extends the knowledge of the relationship between the Na/K ratio and CVD risk, and examines these hypotheses regarding SSBP in association with both the Na: K ratio and CVD risk simultaneously in a large and community-based population.

## 5. Conclusions

In conclusion, the findings from our study elucidate that both SSBP and the Na/K ratio were associated with CVD risk in normotension or stage-one hypertensive individuals. Additionally, the associations between the Na/K ratio and CVD risk can be partially explained by SSBP. Our observations indicate that higher K intake and lower Na intake may help in reducing SSBP and preventing CVD risk in normotension or stage-one hypertensive individuals.

## Figures and Tables

**Table 1:** Subject demographics based on salt sensitivity.

Characteristics	Total (*n* = 2055)	Low-Moderate CVD Risk Group (*n* = 659)	High CVD Risk Group (*n* = 1396)	*p* Value
^a^ Sex (male, %)	555 (27.0)	126 (19.1)	429 (30.7)	<0.001
^b^ Age (years)	59.00 (53.96, 63.29)	53 (48, 58)	61 (57, 65)	<0.001
^b^ BMI (kg/m^−2^)	25.92 (23.84, 28.15)	24.64 (22.66, 26.56)	26.49 (24.59, 28.67)	<0.001
^b^ WHR	0.89 (0.85, 0.93)	0.86 (0.82, 0.90)	0.90 (0.87, 0.94)	<0.001
^b^ FBG (mmol/L)	5.42 (4.99, 6.18)	5.18 (4.86, 5.66)	5.59 (5.06, 6.49)	<0.001
^b^ TG (mmol/L)	1.63 (1.12, 2.48)	1.43 (0.99, 2.07)	1.73 (1.20, 2.64)	<0.001
^b^ TC (mmol/L)	5.04 (4.35, 5.73)	5.03 (4.43, 5.69)	5.05 (4.30, 5.76)	<0.001
^b^ HDL-C (mmol/L)	1.44 (1.13, 2.40)	1.63 (1.23, 2.73)	1.38 (1.09, 2.14)	<0.001
^b^ LDL-C (mmol/L)	2.14 (1.48, 2.86)	2.02 (1.47, 2.75)	2.19 (1.49, 2.93)	<0.001
^b^ 24hUNa (g/day)	3.15 (2.07, 4.50)	3.87 (2.46, 5.78)	4.51 (3.07, 6.36)	<0.001
^b^ 24hUK (g/day)	1.67 (1.14, 2.28)	1.56 (1.08, 2.21)	1.71 (1.18, 2.32)	0.056
^b^ 24hUNa/K	3.17 (2.22, 4.26)	3.08 (2.05, 4.32)	3.21 (2.31, 4.25)	0.060
^b^ Response to acute salt loading, mm Hg				
MAP change 1	0.49 (−4.01, 4.99)	−0.01 (−4.92, 4.67)	1.33 (−2.51, 5.66)	<0.001
^b^ Response to diuresis shrinkage, mm Hg				
MAP change 2	0.33 (−3.83, 4.83)	0.17 (−4.33, 4.83)	1.00 (−3.17, 4.67)	0.398
^a^ Hypertension (*n*, %)	1060 (51.6)	132 (20.0)	928 (66.5)	<0.001
Baseline MAP, mm Hg	92.17 (83.51, 101.01)	83.09 (76.12, 90.51)	95.84 (88.34, 103.84)	<0.001
Antihypertensive medication use (yes) (%)	829 (40.3)	107 (16.2)	722 (51.7)	<0.001
^a^ Diabetes (*n*, %)	352 (17.1)	24 (3.6)	328 (23.5)	<0.001
^a^ Family history of hypertension	1144 (57.8)	350 (53.1)	794 (62.1)	0.010
^a^ Family history of coronary heart disease	532 (25.9)	179 (27.2)	353 (25.3)	0.532
^a^ Family history of stroke	425 (21.5)	137 (20.8)	288 (20.6)	0.871
^a^ Family history of diabetes	527 (26.0)	162 (26.0)	365 (28.4)	0.255
Smoking (yes) (%)	308 (15.2)	80 (12.1)	276 (19.8)	<0.001
Drinking (yes) (%)	962 (47.3)	342 (51.9)	620 (44.4)	0.002

Abbreviations: BMI, body mass index; WHR, waist–hip ratio; FBG, fasting blood glucose; TG, triglyceride; TC, total cholesterol; HDL-C, high-density lipoprotein cholesterol; LDL-C, low-density lipoprotein cholesterol; 24hUK, 24 h urinary potassium excretion; 24hUNa, 24 h urinary sodium excretion; 24hUNa/K, 24 h urinary sodium: potassium ratio; SBP, systolic BP; DBP, diastolic BP; MAP, mean arterial pressure. ^a^ Statistical analysis by chi-square test. ^b^ Statistical analysis by Wilcoxon rank-sum test.

**Table 2:** Association of SSBP with 10-year CVD risk.

SSBP	Univariate Logistic Regression		* Multivariate Logistic Regression	
	OR (95% CI)	*p* Value	OR (95% CI)	*p* Value
Continuous				
MAP change 1, per SD	1.12 (1.11, 1.13)	<0.001	1.03 (1.02, 1.05)	<0.001
MAP change 2, per SD	1.05 (0.94, 1.18)	0.395	1.00 (0.99, 1.01)	0.596
Categories				
MAP change 1, mmHg				
quartile 1 (≤−4.29)	Reference		Reference	
quartile 2 (−4.30 to 0.49)	1.09 (0.80, 1.49)	0.596	1.09 (0.79, 1.49)	0.605
quartile 3 (0.50 to 4.99)	1.34 (0.97, 1.84)	0.072	1.33 (0.97, 1.83)	0.077
quartile 4 (≥5.00)	1.70 (1.23, 2.36)	0.001	1.69 (1.22, 2.33)	0.002
*p* for trend		<0.001		0.001
MAP change 2, mmHg				
quartile 1 (≤−3.83)	Reference		Reference	
quartile 2 (−3.84 to 0.33)	0.71 (0.53, 0.96)	0.025	0.72 (0.54, 0.96)	0.027
quartile 3 (0.33 to 4.83)	0.94 (0.70, 1.28)	0.700	0.94 (0.70, 1.28)	0.708
quartile 4 (≥4.84)	0.90 (0.66, 1.22)	0.486	0.89 (0.66, 1.21)	0.456
*p* for trend		0.915		0.908

* Adjusted for 24hUNa/K.

**Table 3:** Association between sodium, potassium, sodium: potassium ratio, and the 10-year CVD risk.

	Univariate Logistic Regression		* Multivariate Logistic Regression	
	OR (95% CI)	*p* Value	OR (95% CI)	*p* Value
Continuous				
24hUNa, per SD (g/day)	1.13 (1.08, 1.19)	<0.001	1.07 (1.01, 1.13)	<0.001
24hUK, per SD (g/day)	1.10 (1.00, 1.21)	0.054	1.09 (0.99, 1.20)	0.076
24hUNa/K, per SD	1.05 (1.01, 1.09)	0.024	1.05 (1.01, 1.09)	0.025
Categories				
24hUNa (g/day)				
quartile 1	Reference		Reference	
quartile 2	1.57 (1.20, 2.06)	0.001	1.62 (1.25, 2.09)	<0.001
quartile 3	1.54 (1.17, 2.03)	0.002	1.67 (1.29, 2.16)	<0.001
quartile 4	1.720 (1.29, 2.29)	<0.001	1.98 (1.52, 2.58)	<0.001
*p* for trend		<0.001		<0.001
24hUK (g/day)				
quartile 1	Reference		Reference	
quartile 2	1.23 (0.95, 1.59)	0.115	1.20 (0.91, 1.58)	0.204
quartile 3	1.33 (1.02, 1.72)	0.033	1.43 (1.08, 1.89)	0.014
quartile 4	1.44 (1.10, 1.87)	0.007	1.46 (1.10, 1.94)	0.009
*p* for trend		0.006		0.004
24hUNa/K				
quartile 1	Reference		Reference	
quartile 2	1.36 (1.05, 1.75)	0.021	1.45 (1.09, 1.92)	0.010
quartile 3	1.54 (1.18, 2.00)	0.001	1.48 (1.11, 1.96)	0.007
quartile 4	1.49 (1.15, 1.93)	0.003	1.20 (0.91, 1.59)	0.191
*p* for trend		0.002		0.194

* Adjusted for MAP change 1.

## Data Availability

The data that support the findings of this study are available on request from the corresponding author (zlilyepi@ccmu.edu.cn).

## Supplementary Materials

The following are available online at https://www.mdpi.com/article/10.3390/nu14204436/s1: the calculation formula of FRS.
