# Supplementary material for: Higher Potassium Intake and Lower Sodium Intake May Help in Reducing CVD Risk by Lowering Salt Sensitivity of Blood Pressure in the Han Chinese Population
Source: Nutrients. 2022 Oct 21;14(20):4436. doi: 10.3390/nu14204436 (PMC9607620; doi:10.3390/nu14204436)
Supplement: Supplementary file 1 [file nutrients-14-04436-s001.zip › nutrients-1895882-supplementary.pdf]

Supplementary Table S1. Association of SSBP with 10-year CVDs risk by linear regression.

SSBP	Univariate linear regression		* Multivariate linear regression	
	β (95%CI)	P value	β (95%CI)	P value
Continuous				
MAP change 1, per SD	0.22(0.01, 0.43)	0.039	0.29(0.09, 0.49)	0.005
MAP change 2, per SD	0.13(-0.22, 0.48)	0.463	0.11(-0.19, 0.41)	0.467
Categories				
MAP change 1, mmHg				
quartiles 1 (≤-4.29)	Reference	-	Reference	
quartiles 2 (-4.30 to 0.49)	0.44(-0.60, 1.48)	0.405	0.31(-0.73, 1.35)	0.557
quartiles 3 (0.50 to 4.99)	0.35(-0.18, 0.88)	0.190	0.20(-0.32, 0.72)	0.444
quartiles 4 (≥5.00)	0.73(0.35, 1.12)	<0.001	0.70(0.33, 1.08)	<0.001
P for trend		<0.001		
MAP change 2, mmHg				
quartiles 1 (≤-3.83)	Reference	-	Reference	
quartiles 2 (-3.84 to 0.33)	-1.69(-2.62, -0.72)	0.001	-1.49(-2.40, -0.57)	0.001
quartiles 3 (0.33 to 4.83)	-0.54(-1.04, -0.04)	0.033	-0.47(-0.93, -0.01)	0.048
quartiles 4 (≥4.84)	-0.24(-0.59, 0.10)	0.165	-0.32(-0.65, 0.00)	0.053
P for trend		0.318		0.128

*Adjusted for 24hUNa/K.

Supplementary Table S2. Association between sodium, potassium, sodium: potassium ratio, and the 10-year CVDs risk by linear regression.

	Univariate linear regression		*Multivariate linear regression	
	β (95%CI)	P value	β (95%CI)	P value
**Continuous**				
24hUNa, per SD (g/day)	0.48(0.22, 0.75)	<0.001	0.44(0.17, 0.71)	0.001
24hUK, per SD (g/day)	0.35(-0.06, 0.77)	0.094	0.31(-0.11, 0.72)	0.146
24hUNa/K, per SD	0.79(0.34, 1.23)	<0.001	0.82(0.38, 1.26)	<0.001
**Categories**				
24hUNa (g/day)				
quartile 1	Reference		Reference	
quartile 2	2.40(1.28, 3.52)	<0.001	2.31(1.20, 3.43)	<0.001
quartile 3	1.24(0.68, 1.80)	<0.001	1.22(0.66, 1.79)	<0.001
quartile 4	1.13(0.75, 1.51)	<0.001	1.09(0.71, 1.47)	<0.001
P for trend		<0.001		<0.001
**24hUK (g/day)**				
quartile 1	Reference		Reference	
quartile 2	1.37(0.18, 2.55)	0.024	1.29(0.11, 2.47)	0.033
quartile 3	0.28(-0.28, 0.84)	0.33	0.16(-0.40, 0.72)	0.573
quartile 4	0.45(0.06, 0.84)	0.024	0.42(0.03, 0.81)	0.034
P for trend		0.086		0.127
**24hUNa/K**				
quartile 1	Reference		Reference	
quartile 2	1.83(0.65, 3.01)	0.003	1.85(0.66, 3.03)	0.002
quartile 3	1.07(0.46, 1.68)	0.001	1.07(0.47, 1.68)	0.001
quartile 4	0.78(0.35, 1.20)	<0.001	0.80(0.38, 1.23)	<0.001
P for trend		<0.001		<0.001

*Adjusted for MAP change 1.

Supplementary Table S3. Association between sodium, potassium, sodium: potassium ratio, and SSBP stratified by hypertension

	MAP change 1 (mm Hg)				MAP change 2 (mm Hg)			
	[a] With hpertension		[a] Without hypertension		[a] With hpertension		[a] Without hypertension	
	**β (95%CI)**	***P* value**	**β (95%CI)**	***P* value**	**β (95%CI)**	***P* value**	**β (95%CI)**	***P* value**
**Continuous**								
24hUNa (g/day), per SD	-0.240 (-0.512, 0.033)	0.085	0.082 (-0.205, 0.369)	0.576	0.010 (-0.237, 0.257)	0.937	0.325 (-0.341, 0.991)	0.338
24hUK (g/day), per SD	-0.080 (-0.598, 0.439)	0.763	-0.408 (-0.818, 0.001)	0.051	0.152 (-0.726, 1.030)	0.734	0.511 (-0.070, 1.091)	0.084
24hUNa/K, per SD	0.148 (-0.236, 0.531)	0.450	0.576 (0.006, 1.146)	0.048	-0.018 (-0.980, 0.819)	0.860	-0.296 (-1.183, 0.590)	0.512
**Categories**								
**24hUNa (g/day)**								
quartile 1	Ref.		Ref.		Ref.		Ref.	
quartile 2	-0.421 (-1.158, 0.316)	0.262	0.202 (-0.412, 0.316)	0.519	-2.262 (-4.836, 0.313)	0.085	-0.931 (-2.043, 0.181)	0.101
quartile 3	-0.207 (-0.852, 0.438)	0.528	0.399 (-1.635, -0.141)	0.153	-0.675 (-1.901, 0.551)	0.280	0.482 (-0.529, 1.492)	0.350
quartile 4	-0.312 (-0.969, 0.344)	0.350	0.440 (-0.081, 0.961)	0.098	-0.552 (-1.671, 0.568)	0.333	0.000 (-0.406, 0.405)	0.999
P for trend		0.350		0.098		0.569		0.819
**24hUK (g/day)**								
quartile 1	Ref.		Ref.		Ref.		Ref.	
quartile 2	-0.140 (-1.638, 1.358)	0.854	-0.858 (-2.022, 0.306)	0.148	-1.323 (-3.725, 1.080)	0.280	0.583 (-0.543, 1.709)	0.310
quartile 3	-0.517 (-1.151, 0.117)	0.110	-0.645 (-1.171, -0.118)	0.017	-0.586 (-1.736, 0.564)	0.317	0.408 (-0.171, 0.987)	0.167
quartile 4	0.070 (-0.416, 0.557)	0.777	-0.276 (-0.696, 0.145)	0.198	-0.094 (-1.136, 0.949)	0.860	0.537 (-0.164, 1.238)	0.133
P for trend		0.974		0.097		0.855		0.066
**24hUNa/K**								
quartile 1	Ref.		Ref.		Ref.		Ref.	
quartile 2	-0.277 (-1.802, 1.249)	0.721	0.613 (-0.653, 1.880)	0.342	1.846 (0.238, 3.454)	0.025	0.887 (-1.233, 3.008)	0.412
quartile 3	-0.665 (-1.376, 0.047)	0. 067	0.390 (-0.190, 0.970)	0.187	1.226 (-0.609, 3.061)	0.190	-0.315 (-0.950, 0.320)	0.330
quartile 4	-0.186 (-0.726, 0.354)	0.449	0.524 (0.111, 0.937)	0.013	0.598 (-0.511, 1.707)	0.289	-0.225 (-0.658, 0.208)	0.308
P for trend		0.251		0.005		0.332		0.383

[*]Adjusted for age, gender, BMI, FBG, and baseline MAP.
